# Designing and delivering youth mental health services for young people, with young people: what works? A protocol for a realist eDelphi study on effective co-production

**DOI:** 10.1136/bmjopen-2025-105765

**Published:** 2026-05-13

**Authors:** Verity Rose Jones, Imaan Rathore, Justin Waring, Nicola Wright, Sarah-Jane Hannah Fenton

**Affiliations:** 1 Social Policy, University of Birmingham College of Social Sciences, Birmingham, UK; 2 Youth Advisory Group, University of Birmingham, Institute of Mental Health, Birmingham, UK; 3 Social Sciences and Humanities, Loughborough University, Loughborough, England, UK; 4 School of Health Sciences, University of Nottingham, Nottingham, UK; 5 College of Social Sciences, University of Birmingham, Birmingham, West Midlands, UK

**Keywords:** Patient Participation, Adolescent, MENTAL HEALTH, Child & adolescent psychiatry, Community Participation

## Abstract

**Abstract:**

**Introduction:**

Research and mental health services agree that more youth co-production in service design and delivery is needed, but there is little consensus on how to do it well. This study is trying to find agreement about the best ways to do this.

**Methods and analysis:**

A realist eDelphi study will be conducted. People with experience of co-production and engagement in youth mental health services will be invited to participate. This will include young people with relevant lived experience, family members/carers of young people with mental ill health, youth mental health researchers and other professionals with experience of youth engagement work (eg, mental healthcare staff, mental health service managers and participation/engagement professionals in the sector). The target is to recruit 10–20 participants from each of these four groups (40–80 participants total).

The following steps will be taken: (1) an advisory group of young people will use results from a realist literature review completed prior to this study, to generate the first list of items for the survey; (2) using the online survey tool Qualtrics, participants will be invited to rate these items in an online survey. A prompt question formatted using a realist framework will allow participants to comment on their rating and how this survey item works (or does not work) in this context, for young people or other stakeholders. Participants will be able to add further suggestions at this stage; (3) using Qualtrics, a second survey round will be completed which will include the new suggestions from participants, and original items with their average participant ratings and comments displayed. Participants will be asked to rerate items in this round; (4) a list of items will be generated that comprises survey items believed to be ‘essential’ or ‘important’ by 80% or more of the participants; (5) this list will be discussed with the Youth Advisory Group to generate a final recommendations document and consider creative outputs and dissemination methods. Data analysis will include raw numbers, means and frequencies.

**Ethics and dissemination:**

Ethical approval has been granted by the University of Birmingham Research Ethics Committee (Ref: ERN_1550-Apr2024). Both traditional and non-traditional outputs will be created (eg, conference presentations, publications, a plain English summary and social media infographics).

**STRENGTHS AND LIMITATIONS OF THIS STUDY**

- This is to our knowledge the first research to adopt realist principles into a Delphi study and incorporate extensive patient and public involvement (PPI) with young people in the realist Delphi design and analysis.
- Innovatively combining the realist approach with a Delphi method means the three project elements (stakeholder workshops, rapid realist literature review and Delphi survey) will provide robust, comprehensive findings.
- The literature review and Delphi survey will be conducted in English only.
- The sampling method, while effective, may miss some relevant participants not linked to the identified networks.
- This is an untested innovation in relation to established methodology—it is a hybrid of realist principles and Delphi methods—as such it may not work.

## Introduction

 Mental health services across the globe are unable to meet the needs of young people, and there are tensions wherein services themselves can cause harm and if misdirected do not offer much needed support.^1^,^3^ The argument is increasingly made that service users know their needs best, and thus involving young people with lived/living experience of mental illness and distress in designing and delivering mental health services would mean this knowledge could be leveraged through co-production to improve these services.^4^,^6^

Co-production is often considered the ‘gold standard’ of involvement practices, and policy and practice guidelines increasingly recommend using co-production in youth mental health services.^46^,^8^ Co-production is described by the National Co-production Advisory Group as:

Co-production is an equal relationship between people who use services and the people responsible for services. They work together, from design to delivery, sharing strategic decision-making about policies as well as decisions about the best ways to deliver services.^9^

Though research and mental health services agree that we should be doing more co-production and youth engagement, there is little consensus on exactly what constitutes gold standard co-production nor how to do it well.^10 11^ To understand this further, the authors completed a rapid realist literature review to provide an overview of evidence specific to how co-production works in youth mental health services.^12^ This review was deemed to be necessary as existing literature reviews in this area either do not focus on youth^6^ on co-production specifically,^13^ on co-production of services,^14 15^ or do not use a review method which takes a realist approach to identify ‘what works?’^11^ and thus exclude much relevant literature.

The review extracted data from 57 papers which contributed to five context-mechanism-outcome (CMO) configurations to describe the generative mechanisms by which co-production in youth mental health services is linked to outcomes and influenced by context (see Methods section for further information on the realist approach and terminology). The final programme theory which emerged from the review about how and why co-production methods in youth mental health services work, for whom and in which circumstances requires further validation and input from stakeholders to be developed into practice guidelines or practical tools that can be used in the real world. This is the aim of the realist e-Delphi study proposed in this protocol. The Delphi technique is recommended for use when empirical evidence is limited^16^ as is the case for this topic.^11^

The proposed realist e-Delphi study is trying to find agreement about the best ways to do youth engagement and co-production by asking the question: When designing and delivering mental health services for young people, with young people—what works? It is this emphasis on the how, for whom, in what circumstances and why that we feel justifies the adoption of realist principles into Delphi methodology. Realism itself allows for *both* subjective and objective realities to coexist and this is helpful when integrated with a lived experiential reality, as this grounded experiential reality allows us to test potentially subjective individual experiences against those broader experiences of young people to try and get as close to an objective reality (without denying subjective experience) as possible. To our knowledge, this will be the first attempt to bring together realist principles, Delphi methodology and co-production with those with lived experience.

The key benefit of this research will be the production of a consensus from stakeholders of youth mental health services (service users, professionals, researchers and families/carers) on principles for effective co-production in youth mental health services. This will be achieved by sending a list of statements to a wide group of expert stakeholders in a survey. There will be two rounds of voting to prioritise these statements according to the expert stakeholder participants by rating their importance on a Likert scale. Participants can add to the list of statements through the online survey in round one and prioritise these new statements alongside the original list of statements in round two. Best practice tips and guidelines for using the Delphi technique in health science research will be followed.^17 18^ The expected outcome of this Delphi survey is a list of principles for effective co-production/youth involvement in the context of youth mental health services.

The resulting list will be relevant to young people and professionals using, or intending to use, involvement practices within these settings and to researchers and policy makers in the sector. The results of this consensus study will be also used for a realist action research project aiming to use co-production to facilitate quality improvement in youth mental health services, thereby testing the results from this Delphi study in a real-world setting.

**Aim:** to develop a consensus-based prioritised list of the most important considerations for achieving effective co-production/youth engagement in youth mental health service design and delivery.

## Methods and analysis

### Study overview

Prior to the Delphi survey, a rapid realist literature review and a series of eight stakeholder workshops (see table 1) have been completed to identify a long list of survey items (see online supplemental appendix 1). Demographic information for participants in these workshops was not collected.

**Table 1:** Stakeholder workshops

Date of workshop	Participants	Group	Purpose of workshop
March 2023	6 young people aged 18–25, 2 participation professionals	IMH YAG	Present project, identify research question/research priorities, brainstorm/identify ‘what works’ for co-production in youth mental health, review patient and public involvement (PPI) strategy.
July 2023	4 young people aged 16–25, 1 participation professional	Think4Brum	Present project, gather feedback on research question/focus and PPI strategy.
August 2023	10 researchers (youth suicide prevention research)	Researchers	Present project, brainstorm/identify ‘what works’ for co-production in youth mental health (both services and research), review PPI strategy.
November 2023	5 young people aged 18–25, 1 participation professional	IMH YAG	Review documentation for Delphi study ethics, refine initial programme theory (presented as ‘if/then/because’ statements).
November 2023	4 young people aged 16–25, 1 participation professional	Think4Brum	Refine initial programme theory (presented as ‘if/then/because’ statements).
February 2024	7 young people aged 18–25, 1 participation professional	IMH YAG	Present provisional literature review findings, rate agreement with CMO statements/discuss, develop concept map, discuss draft of final programme theory, review recruitment poster suggestions for Delphi study.
April 2024	7 young people aged 18–25, 1 participation professional	IMG YAG	Review literature review results and develop into survey items for Delphi study (reduce/remove jargon, ensure each item makes sense, add additional ideas). Final edits to programme theory.
April 2024	5 young people aged 16–25, 1 participation professional	Think4Brum	Present literature review results and gather feedback, discuss next steps.

CMO, context-mechanism-outcome; IMH, Institute for Mental Health; YAG, Youth Advisory Group.

**Figure 1:**
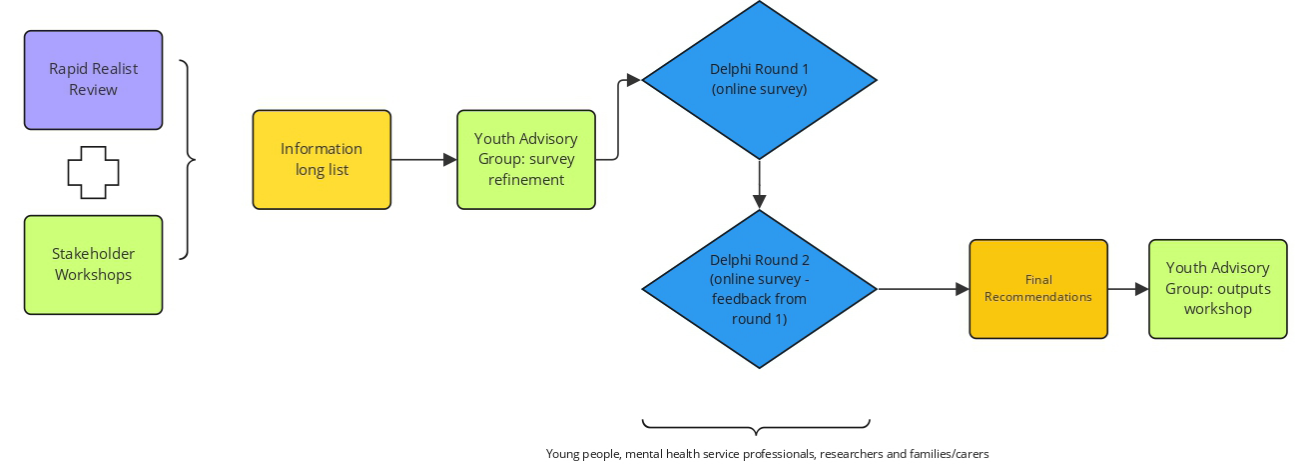
Study overview diagram.

The proposed study will follow a Delphi process but has adapted this to undertake a ‘realist Delphi’ in which participants will add to and rate this list of items to identify consensus on effective practices for co-production and involvement with young people in mental health service design and delivery. See figure 1 for stages of the study and planned stakeholder input.

#### Patient and public involvement

Patients and/or the public were extensively involved in the design, conduct, reporting or dissemination plans of this research. The University of Birmingham Institute for Mental Health (IMH) Youth Advisory Group (YAG) is a group of young people who advise on mental health research. They chose the research topic and question, helped to develop the survey questions, edited the research documents and will input into the analysis of the results and decisions of how to disseminate these. In addition, a youth peer researcher will collect and analyse the data alongside the lead researcher. A young public advisor also gave advice on how young people were involved in designing the study. The UK standards for public involvement from the National Institute for Health Research will be followed throughout including reimbursement at or above National Institute for Health and Care Research (NIHR) standard rates.^19^

### The realist approach

Realist research is concerned with finding answers to real-world questions and is often framed through the enquiry ‘what works, for whom, in what circumstance?’.^20 21^ Using this lens requires researchers to identify causal links between contexts, generative mechanisms (see table 2) and how these two interact to produce outcomes. For example, a participatory-realist evaluation of peer support for young people coping with complex mental health and substance use challenges^22^ identified the CMO:

C (Peer supporters share similar experiences and recovery journeys with clients) + M (Peers demonstrate positive identity and wellness while moving forward in recovery & clients develop a more positive evaluation of shared social reference group) → O (Clients experience enhanced positive identity, decreased self-stigma and enhanced wellbeing).

**Table 2:** Glossary of realist terminology

Realist terminology	Definition
Realism	The philosophical position that though objective reality is independent of us, it can only be understood through human interpretation and perception (sometimes described as sitting between positivism and constructivism).^40 41^
Context	The backdrop of programmes: pre-existing conditions (in the physical/social environment) which influence the success or failure of different interventions or programmes.^20 21 42^
Generative mechanism	Stakeholders’ response(s) to resources offered, the underpinning generative/causal force(s) that leads to outcomes, these forces themselves are not observable. For example, these could be trust, participant engagement, the organisational culture and information shared.^20 21 42 43^
Outcome	Intended and unintended consequences of a mechanism operating within a context. The measurable impact at the behavioural, clinical or system level.^20 21 42^

The benefit of a realist approach is that it aims to clearly identify the often unseen or invisible causal link (mechanism) between the context and the outcome.

Realist principles have been applied to this study in three ways:

1. First, the research question under investigation uses realist framing: “When designing and delivering mental health services for young people (i.e. *in what circumstances?*), with young people (i.e. *for whom*) – *what works*?”.
2. Second, the underpinning data drawn from the literature review are presented as CMO configurations that itself adopted a realist methodology. These data were then developed into the survey items with partnership with the YAG.
3. Third, (and untested methodologically to our knowledge) the prompt question within the survey itself asks participants to make causal connections in their responses as it follows the same realist formula in the question: “Why have you ranked this statement in this way, tell us why it should be included/excluded? (e.g. Who does this work for? In what circumstances does this work/not work?)”.

### Participants and sample size

Participants will be selected because of their experience designing or delivering mental health services for young people. To take part, they are required to have engaged in at least one co-production/youth involvement project designing or delivering mental health services for young people and will be asked to self-identify their role within this work. Given the inconsistency in applying terminology around co-production, and the requirement of participants to self-identify, we felt this might result in the exclusion of those who do not describe their work in this way and so to mitigate this we have asked participants to choose a term themselves to describe the work they were involved in, that is, co-production, youth involvement, youth engagement and other. Second, they will be asked to respond to three questions describing the work to help identify if it fits the definition of co-production used here, that is,

- Were young people involved from the very start of the project?
- Did they young people involved have equal decision-making power?
- Were they included in decisions on all relevant topics?

We will further add a free text box for participants to briefly describe the project to enrich this and enable further reporting on the types of engagement/involvement and co-production captured. This will increase the robustness of validity checking to try and ensure we do not get ‘ghost’ or fake participants.

To gather a wide range of stakeholder input, participants will be drawn from four groups with the aim to recruit 10–20 participants per group (40–80 participants total). Keeney *et al*^17^ identify that there is no guidance on the minimum or maximum number of experts required and advise using rigorous inclusion and exclusion criteria. Accordingly, participants must have real-world experience of co-production or engagement work designing or delivering mental health services for young people to take part, and be from one of the four groups of experts who comprise the target population:

- Youth: young people aged 11–25^23^ with lived or living experience of mental illness/distress
- Professionals: mental health professionals, service managers, participation/engagement professionals
- Researchers: consumer or lived experience academics, specialist researchers in youth mental health
- Families/carers: family members/carers of young people with lived or living experience of mental illness/distress

Recruitment will be global through three channels. First, recruitment emails sent to existing networks of the research team, which is becoming best practice in relation to online recruitment^24^ to try and limit the number of fake or ‘ghost’ participants. Second snowball sampling of these email invitations and finally volunteer sampling via social media posts (on LinkedIn, Twitter/X, Bluesky and Instagram). These sampling methods are suitable as the target population is difficult to identify^25^—in this case, there are no centralised registries or dedicated organisations made up of people who have engaged in co-production work in youth mental health services, so combining volunteer and snowball sampling are the best routes for finding the target population. The recruitment email and social media posts will provide a link to access the information about the study and the consent form. Participants from all countries can take part, though all study information will be provided in English. Efforts to recruit diverse panel members from minoritised groups will include the following: inviting international authors from papers in the global literature review that precedes this Delphi study and sending invitations via networks targeting minoritised or specialist groups. For example, these groups will include McPin Foundation, international co-production collectives, and mental health and social justice networks. Study participants will receive a £25 token after completing both rounds of the survey.

In anticipation of the cyber threat to online research of survey bots or insincere respondents, a number of safeguards will be in place in this Delphi survey, namely, multiple free text boxes, ReCAPTCHA technology, Qualtrics BOT detection, a signature required for consent, demographic data collected and cross-verified from repetitive questions or between surveys, and online recruitment to be shared only on private social media channels. Nevertheless, it is now almost impossible to avoid insincere participants in online research;^26 27^ accordingly, this study will integrate rigorous data cleaning to identify valid responses if required.^28^

### Initial topic generation/development of the questionnaire

The initial topics for the Delphi rounds were generated through two methods: a rapid realist literature review and stakeholder workshops (see further detail in online supplemental appendix 1). This was to ensure that round one of the survey includes recommendations from the best available research evidence as well as from both experts by experience and experts by training who contributed via the workshops and literature review expert panel. It is beneficial to combine these different types of knowledge in research methods where possible^15 29^ as well as being best practice in realist methodology to maximise stakeholder involvement in knowledge generation.^30 31^

#### Drawing on the existing literature review

**Figure 2:**
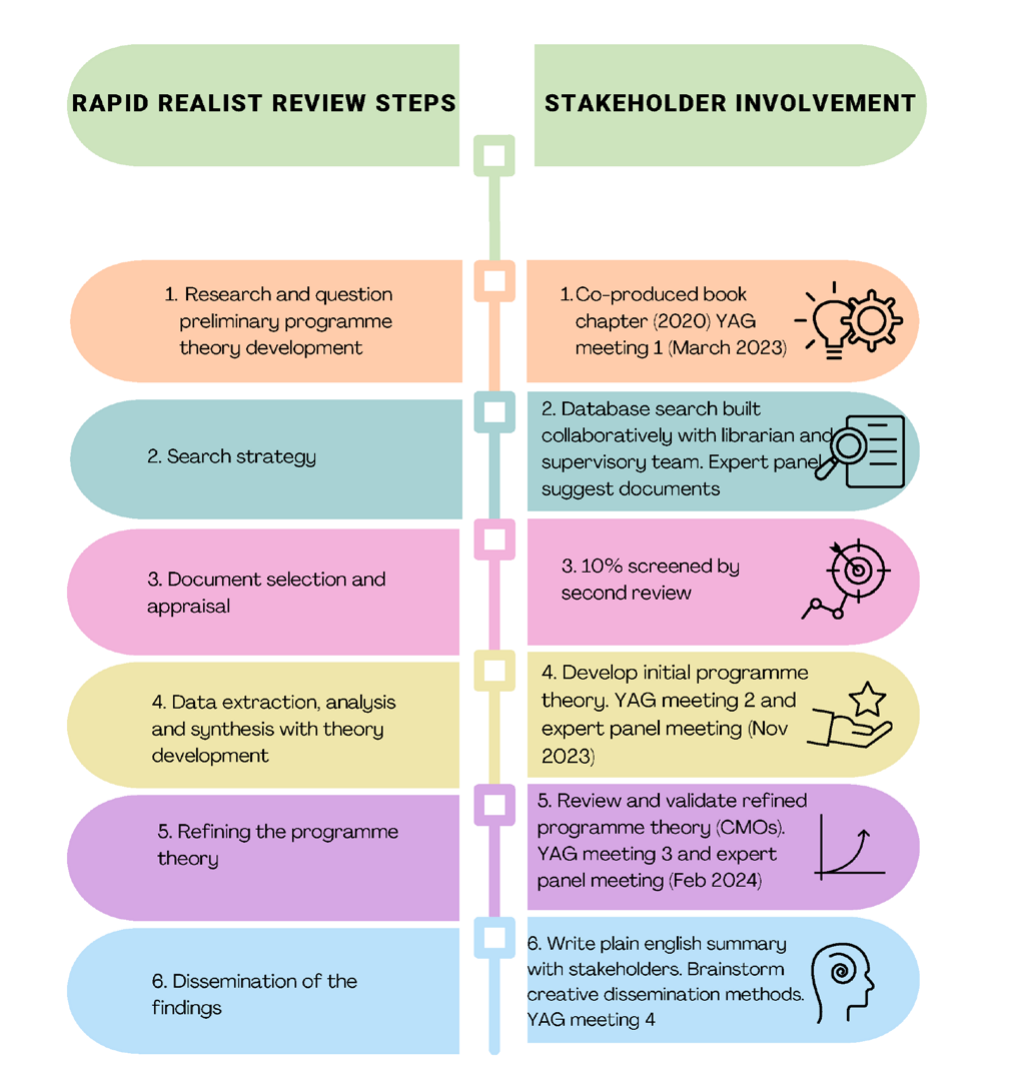
Stakeholder involvement in the literature review: visual timeline for stakeholders of review activity and parallel involvement. CMOs, context-mechanism-outcomes; YAG, Youth Advisory Group.

A rapid realist review of relevant documents (academic and grey literature) was completed which synthesised evidence to develop CMO configurations that can inform policy and practice. Stakeholders were iteratively involved by engaging an expert panel including young people and separate YAG (see figure 2).

Full results have been reported according to the Realist And Meta-narrative Evidence Syntheses: Evolving Standards (RAMESES) .^12 32^ Data extracted from 57 papers contributed to five CMO configurations to describe the generative mechanisms by which co-production in youth mental health services are linked to outcomes and influenced by context. See table 3 for the programme theory with five CMOs that emerged from the literature review.

**Table 3:** Programme theory

*Research question*	For whom and in what circumstances does co-production work in youth mental health services?	
*Key generative mechanisms*	*1. A supportive organisational culture enabling youth experts by experience (YEBE) to be perceived as credible knowers.* *2. YEBE authentically participating as co-producers and benefiting from personal development (vocational skills, psychological wellness and new knowledge).* *3. Transparency about limits to the scope for change producing trust and shared understanding.* *4. Wider stakeholders sharing power and responsibility with YEBE leading to mutual respect, trust and stakeholders listening to one another.* *5. Programmes having sufficient resources and being flexible to new ways of working enhances accessibility for participation from minoritised groups*.	
*Final programme theory*	*YEBE, particularly those from minoritised communities, provided with a supportive organisational culture can experience authentic engagement where their knowledge is perceived as credible by wider stakeholders. This leads to personal development for participating YEBE as well as service improvements from their input*.	(*whom*)
		(*in what circumstances*)
		(*co-production works*)

Although the findings from this review indicate that key to effective co-production in this context is a ‘supportive organisational culture’, questions remain about what this comprises in real-world services aiming to do effective co-production. This Delphi survey aims to build on this existing programme theory and develop and refine it further by seeking consensus about what the tangible actions are that constitute (a) sufficient resourcing, (b) flexible working practices and (c) stakeholders sharing power with young people.

#### Stakeholder workshops

As required for a rapid realist review, stakeholders were invited to feedback on the theories which emerged from the literature to help develop preliminary programme theory, which fed iteratively into the CMO configurations.^30^ These workshops were with two groups of young people (the IMH YAG and Think4Brum) and specialist researchers. In addition to these workshops, participation staff from a range of organisations were consulted during a Turing scholar visit to Melbourne, Australia, or through expert panel participation. Further detail on these workshops is available in the literature review protocol.^33^

As well as workshops to generate theory during the literature review, a workshop was conducted with the IMH YAG to refine the survey itself. This involved considering each survey item for unnecessary jargon, checking the meaning is clear and identifying any missing items to add to the long list (see online supplemental appendix 1). Finally, following completion of the Delphi survey, a workshop to consider Delphi results and choose or develop research outputs will take place with the YAG. See figure 2 for how these workshops feed into the overall research stages and table 1 for an overview of the workshops.

### Online survey

#### Providing consent/demographics

The online survey platform Qualtrics^34^ will be used. Prospective participants will be provided with a participant information sheet on Qualtrics, detailing what participation will involve, how their information will be stored and the right to withdraw. A digital consent form on Qualtrics will be used. Once the consent form has been completed, participants will be asked for demographic information and then have access to the survey. Demographic information collected will include the following: current location, age, gender identity, role(s) relevant to the study, lived experience of mental distress/illness (yes/no/prefer not to say), carer to someone with lived experience of mental illness/distress (yes/no/prefer not to say), identification with one or more protected characteristic (not required to specify which), location of the co-production project/programme(s) they were involved in. For sensitive data, participants will be able to select ‘prefer not to say’.

Age eligibility for participation will be that of UK secondary school age and older (11 years). In accordance with UK law, any participants under the age of 16 will require written consent from an adult with parental responsibility to participate. This will be via use of an online consent form on Qualtrics on which parents/carers are required to confirm consent, provide their name and a digital signature. Parents/carers will be provided with the study details and participant information sheet before giving consent. Following the innovative methods of the trailblazer study, participants over the age of 16 will be able to self-consent.^35^ Participants will be informed that they can withdraw participation from the study at any time without giving a reason, that they can withdraw their responses for up to 2 weeks after submitting them and that there are no consequences to them for withdrawing. The survey will take place in two rounds. There is no set standard for Delphi rounds (usually this is guided by reaching consensus); however, as we are starting with evidence drawn from the literature review that was refined in partnership with young people, some work towards consensus building prioritising those with lived experience has already taken place. We have replaced a round with this initial activity to try and mitigate participant burden (particularly with young people and busy professionals) and consequent attrition. We will, however, if consensus is not reached consider a third round. Limiting the survey to two rounds is usual practice for a modified Delphi where round one is replaced by workshops, focus groups or reviewing the literature and the initial list of survey items is generated through these activities.^18^ The process for generating this initial list is described in the next section. A similar two-round process was used in a recent modified eDelphi in youth mental health focused on suicide research.^36^

#### Round one

The statements on the Delphi survey will comprise findings from the literature review and the preliminary programme theory developed with the YAG, expert panel and specialist researchers. The YAG will meet to identify the full set of survey questions. Statements will be grouped under topic headings linked to the literature review findings:

- How can wider stakeholders share power and responsibility with youth experts by experience?
- What comprises sufficient resources?
- What does flexibility to new ways of working look like?
- What else is important?

Each statement will be an action that can be taken by stakeholders doing co-production work in mental health services for young people. Some examples of these action statements from the scoping exercises and literature review are the following:

- Invite young people to co-produce on all topics.
- Involve young people from the outset.
- Provide training on co-production for all stakeholders.
- Create an informal environment.

In accordance with standard Delphi process,^17 18^ the participants will be presented with a Likert scale to rate each statement for inclusion in a best practice checklist with the options:

1. essential, 2. important, 3. unimportant, 4. should not be included.

Underneath each statement, there will be a free text box where participants can comment on why they have ranked each statement at their chosen point on the scale. The prompt for this box will state “Why have you ranked this statement in this way, tell us why it should be included / excluded? (e.g. Who does this work for? In what circumstances does this work / not work?)”. This prompt uses a realist framing to encourage participants to identify causal connections between what works and the specific circumstances found in youth mental health services with this population.^20 37^

Criterion for inclusion in the final research output is an a priori set consensus level of at least 80% agreement that the statement is ‘essential’ or ‘important’. Keeney *et al*^17^ suggest 75% as a minimum level with 80% as best practice. 80% has been used in other recent Delphi surveys within youth mental health research.^38 39^ The survey will be piloted by members of the research team to check for clarity and functionality. As is usual for Delphi studies in the round one survey, participants will also be invited to add new statements if they feel anything is missing from the list of statements.^18^

#### Round two

In round two of the survey, participants will receive a list of statements to rank on the Likert scale, as in round one. Qualitative comments from participants for or against inclusion for each statement will also be included anonymously alongside the percentage agreement each proposal achieved in round one (both overall agreement and level of agreement from each group will be shown). This is so that participants can consider the perspectives of other contributors when re-rating, therefore maximising consensus. The list of statements sent in the round two survey will include the following:

- Any new statements suggested by survey participants during round one (similar statements will be combined).
- Statements which received 20%–79% agreement of being ‘essential’ or ‘important’ as ranked by all participants.
- Statements with less than 20% overall agreement but which 80% of the youth panel endorsed as ‘essential’ or ‘important’.

All other statements ranked in round one which received less than 20% agreement across all participant groups of being ‘essential’ or ‘important’ will be excluded from the round two questionnaire. Less than 20% agreement is routinely used as a cut-off for consensus in Delphi studies.^17 38 39^ This process will be done collaboratively by the lead researcher and young peer researcher.

At the end of round two, a list of statements that have reached consensus will represent the results of the study. Relevant comments from panellists will be presented alongside this list in the study write-up, intended for publication in an academic journal. See the Data analysis and anticipated outputs section below for further anticipated outputs.

### Security of the data

All records obtained during this study will remain strictly confidential, within the limits of the law. Only the researchers and those employed on the study will have access to the data, which will be stored on secure servers with only information necessary for the research study being collected. Participants will not be identified in any report or publication that is produced. Where quotations from survey responses are shared in round two or in publications, these will be anonymised. The data will be held for a maximum of 10 years in line with University of Birmingham guidelines, and then they will be destroyed.

### Data analysis and anticipated outputs

This research is scheduled to take place from autumn 2025 to spring 2026. Participants will receive a lay summary of the study results as well as the results in full. If the study is successfully published, then a link to any publications will be circulated to everyone who takes part. Participants will also receive a debrief email. The lead researcher will meet with the YAG to discuss creative dissemination of the results, this could include devising a practice checklist, video, infographics or other outputs that they feel would best reach the desired audience of stakeholders in youth mental health service co-production and engagement work.

### Conclusion, strengths and limitations

There are several strengths in bringing together Delphi methodology, lived experience and realist methods—if successful, this approach will allow us to answer a broader range of questions using Delphi methodology, while also helping to flatten the hierarchies that often plague traditional Delphi approaches. However, in doing something new, there are risks that it will not work and there will be other issues raised by our recruitment strategy that will fail to reach significant groups of young people and professionals, particularly those who have had bad experiences. We have attempted to mitigate this by drawing on a wide range of empirical evidence in the foundations of the statement building; however, we accept that no method is perfect and we will seek to address this in the third study wherein we take ‘real world’ empirical research.

Another potential concern for recruitment is that the minimum participation requirement—involvement in at least one piece of participatory work designing and delivering mental health services for young people—may admit participants with limited experience. However, given that experience in this area is often both undersold and exaggerated, an inclusive recruitment strategy will be adopted, prioritising transparent reporting over exclusion. Participants will therefore be asked to describe their project(s), self-report their position on co-production ladders and assess their work against the three elements of the co-production definition used in this study. This allows the quality and depth of participants’ experience to be reported, while avoiding the exclusion of those who have valuable insight despite having limited experience—such as young people involved in single short-term projects, or those who are overly modest about their participatory work.

This study is also limited in its heavily UK-centric/high-income countries-centric approach both in available networks to recruit from and the use of English language only—however—it is a pilot, and if successful, it will allow other researchers in different contexts to adopt similar approaches to broaden the international evidence base. There will be likely wide heterogeneity of approaches in the participants surveyed (mirroring the wider literature) and consequently we are not looking for examples of similar work, but we are seeking the conditions in which that work was successful and how that was experienced in order to understand what generalisable learning can be gleaned. It is not an exhaustive methodology and this is a limitation of this kind of study; however, the object of interest is co-production in youth mental health services, which are in and of themselves diverse, and we are explicitly seeking to explore this complexity.

## Ethics and dissemination

Ethical approval has been granted by the University of Birmingham Research Ethics Committee (Ref: ERN_1550-Jul2024). The findings are intended to be published in a peer-reviewed specialty journal and presented at relevant national and international conferences. In addition to these traditional methods a plain English summary will be devised with the youth advisory board alongside a dissemination strategy for reaching real-world audiences of interested stakeholders. The aim is to disseminate results and information as broadly as possible.

## Supplementary material

10.1136/bmjopen-2025-105765 online supplemental file 1
