# Supplementary material for: Designing and delivering youth mental health services for young people, with young people: what works? A protocol for a realist eDelphi study on effective co-production
Source: BMJ Open. 2026 May 13;16(5):e105765. doi: 10.1136/bmjopen-2025-105765 (PMC13182383; doi:10.1136/bmjopen-2025-105765)
Supplement: online supplemental file 1 [file bmjopen-16-5-s001.docx]

Appendix 1: Survey Items Long list

| **SHARING POWER** |
| --- |
| Agree shared goals at the outset |
| Young people are invited to co-produce on all topics |
| Share results of the work (service improvements) with everyone |
| Clear aims |
| Young people are present when decisions are made |
| Include in delivery / to the end of the programme |
| Young people are involved in the decision making process |
| Ensuring young people outnumber adults |
| Redress power imbalances |
| Acknowledging and minimising power differentials |
| Clear communication between meetings - ask for communication preferences (eg email, whatsapp) |
| Involve young people from the outset |
| Involve young people throughout |
| Be honest about the limits of the project /context |
| An age bracket for applying |
| Have more than one young person contributing to the co-production group/project |
| **SUFFICIENT RESOURCES** |
| money! |
| time to plan co-production / engagement work thoroughly |
| Minutes or notes from the meeting or like access to presentations / talk shared with all participants |
| Money to pay young people |
| Mentorship provision (for young people) |
| Psychological and pastoral support available for all co-producers (young people and other stakeholders) |
| dedicated staff time to plan, implement and conduct co-production? |
| Training on co-production for all stakeholders |
| Clear point of contact |
| Spaces to meet |
| Recognition of stakeholder contributions (e.g. payment, named author, thanked publicly, speaking at events) Tailored to YP interests |
| appropriate rates of pay |
| participation roles (staff who work full-time on engagement) |
| Snacks and drinks |
| **FLEXIBLE WORKING** |
| Using quotas to recruit young people with different experiences / identity characteristics to best represent the community |
| Inclusion practices (e.g. pronouns, trigger warnings, recruitment) |
| Not cherry-picking / censoring recovery stories |
| Changing the times of meetings to outside of 9-5 |
| Cultural competence (discussion of racism / discrimination) |
| Slowing the working processes to meaningfully listen |
| Creating an informal environment |
| Limiting jargon |
| Providing support for the application process |
| Asking at the outset ‘is co-production really appropriate here?’ |
| Offering a variety of roles for young people (not just those which require ‘professional’ skills such as public speaking/ attending meetings) |
| Holding meetings online / hybrid |
| Providing training to ensure shared understanding of co-production |
| Use quotas (ethnicity, age, other protected characteristics) when recruiting, to make sure the youth involved represent the target community of the service |
| **TRANSPARENCY ABOUT LIMITS TO THE PROJECT SCOPE** |
| No actions identified during the review |
