# Supplementary material for: Designing and delivering youth mental health services for young people, with young people: what works? A protocol for a realist eDelphi study on effective co-production
Source: BMJ Open. 2026 May 13;16(5):e105765. doi: 10.1136/bmjopen-2025-105765 (PMC13182383; doi:10.1136/bmjopen-2025-105765)
Supplement: Supplementary file 2 [file bmjopen-2025-105765.reviewer_comments.pdf]

PEER REVIEW HISTORY

BMJ Open publishes all reviews undertaken for accepted manuscripts. Reviewers are asked to complete a checklist review form and are provided with free text boxes to elaborate on their assessment. These free text comments are reproduced below.

ARTICLE DETAILS

Title (Provisional)

Designing and delivering youth mental health services for young people, with young people – what works? A protocol for a realist eDelphi study on effective co-production.

Authors

Jones, Verity Rose; Rathore, Imaan; Waring, Justin; Wright, Nicola; Fenton, Sarah-Jane Hannah

---

VERSION 1 - REVIEW

**Reviewer**	**1**
**Name**	**Gutman, Leslie**
**Affiliation**	**University College London**
**Date**	**23-Jan-2026**
**COI**	**None**

Thank you for the opportunity to review this article again and to provide further comments.

Thank you also to the authors for considering and responding to reviewers' previous comments. I think that the article is close to being ready for publication but there are still some areas that I believe would benefit from further revision. Some of these comments I think may have been made in the previous version but have either not been addressed or reasons for not addressing have not been provided:

- In a couple of places sentences are incomplete or are phrases making them grammatically incorrect - for example page 5 line 34 and page 7 line 14

- Page 8 discusses the need for rigorous inclusion and exclusion criteria but then uses the word "some" to describe the experience required for experts. The word some is rather loose and open to interpretation and doesn't appear to show rigour. Could you expand on this and describe more specific criteria for determining whether someone has sufficient experience to be part of the panel

- On page 8, line 40 you refer to recruiting participants from any country. What demographic data do you intend to collect about participants? Will you be making efforts to recruit diverse panel members from the minoritised groups mentioned in line 3 of page 9?

- In figure 2 box 3 a capital D is needed for consistency

- On page 10 line 30 please provide more information about the parental consent process. What does the opt-in mean in practice?

- Page 10 line 43 I think this should read the youth advisory group will "meet" rather than "met"

- As you are using social media to advertise for participants, how will you verify that these are real people and ones who meet your eligibility criteria?

- Page 11 line 12, there is no option 3 listed - this appears to be an omission.

- It is still not clear why the Delphi study will be only two rounds rather than the usual 3. This approach just needs to be explained and justified (perhaps citing a previous study that takes this approach)

- At the end of page 11 I feel there is a line missing - what will the final output be? I presume that at the end of Round 2 you will present a list of statements that have reached consensus- this will represent the final results of the study. Is that correct? Will you report any final comments by panellists or just the list? This could be more explicit

- A short conclusion could be helpful here - identifying some of the limitations of the study, for example the English language only option for participants which is mentioned earlier.

VERSION 1 - AUTHOR RESPONSE

Comment	Response
REVIEWER 1	
Thank you also to the authors for considering and responding to reviewers' previous comments. I think that the article is close to being ready for publication but there are still some areas that I believe would benefit from further revision. Some of these comments I think may have been made in the previous version but have either not been addressed or reasons for not addressing have not been provided.	Thank you for the supportive comment that the article is close to being ready for publication. Thank you also for the helpful constructive comments each comment has been carefully addressed in this table.
- In a couple of places sentences are incomplete or are phrases making them grammatically incorrect - for example page 5 line 34 and page 7 line 14	Thank you for highlighting these errors, these have been corrected, see: introduction paragraph 5 and opening sentence of section 8. In addition, the whole manuscript has now been thoroughly proofread to check for grammatical and spelling errors.

Comment	Response
- Page 8 discusses the need for rigorous inclusion and exclusion criteria but then uses the word "some" to describe the experience required for experts. The word some is rather loose and open to interpretation and doesn't appear to show rigour. Could you expand on this and describe more specific criteria for determining whether someone has sufficient experience to be part of the panel	Thank you for highlighting the lack of specificity in the use of the word 'some' here. Criteria required for joining the panel are now clearly described in this section. In addition, clarity has been added in the first paragraph of the 'participants and sample size' section that participants need real-world experience of participatory work designing or delivering mental health services for young people to take part. The word "some" has been removed.<br><br>An inclusive recruitment strategy will be adopted, prioritising transparent reporting of participants experience over exclusion - a discussion of the rationale for this approach and it's limitations has also been added to the 'conclusion, strengths and limitations' section.
- On page 8, line 40 you refer to recruiting participants from any country. What demographic data do you intend to collect about participants? Will you be making efforts to recruit diverse panel members from the minoritised groups mentioned in line 3 of page 9?	Thank you for highlighting this oversight. Demographic information which is intended to be collected is now listed under the section heading 6.1 "Providing Consent / Demographics".<br><br>Details regarding efforts to recruit diverse panel members has now been provided under section 4 'Participants and Sample Size'.
- In figure 2 box 3 a capital D is needed for consistency	Thank you, this has been amended.
- On page 10 line 30 please provide more information about the parental consent process. What does the opt-in mean in practice?	Details of this process have been added and the word 'opt-in' removed to avoid confusion. Parental /carer consent is required for all participants under 16.
- Page 10 line 43 I think this should read the youth advisory group will "meet" rather than "met"	Thank you, this has been amended.
- As you are using social media to advertise for participants, how will you verify that these are real people and ones who meet your eligibility criteria?	This is very important and we thank the reviewer for noticing this omission. The safeguards in place and use of rigorous data cleaning if required are now described at the end of section 4.
- Page 11 line 12, there is no option 3 listed - this appears to be an omission.	Many thanks, the misnumbering has been amended.
- It is still not clear why the Delphi study will be only two rounds rather than the usual 3. This approach just needs to be explained and justified (perhaps citing a previous study that takes this approach)	Many thanks for highlighting the lack of explanation here. Further explanation about the two-round process in modified Delphi, where round one is replaced with workshops (in this case 8 workshops and a

Comment	Response
	literature review) is now provided. References have been added for both a recent previous study in the same field which takes this approach and a methods textbook which outlines the 2-round modified Delphi process where round 1 is replaced in this way.
- At the end of page 11 I feel there is a line missing - what will the final output be? I presume that at the end of Round 2 you will present a list of statements that have reached consensus- this will represent the final results of the study. Is that correct? Will you report any final comments by panellists or just the list? This could be more explicit	Thank you for pointing out this omission. This has been added at the end of section 6.3.
- A short conclusion could be helpful here - identifying some of the limitations of the study, for example the English language only option for participants which is mentioned earlier.	Thank you, the "strengths and limitations" section has been updated to be titled "Conclusion, Strengths and Limitations" and now also includes discussion of limitations flagged by the reviewer here:<br>- The survey's availability in English language only<br>- Possibility that some participants may have only a small amount of experience of co-production in this field (e.g. young people involved in one short project)

VERSION 2 - REVIEW

**Reviewer**	**1**
**Name**	**Gutman, Leslie**
**Affiliation**	**University College London**
**Date**	**24-Apr-2026**
**COI**	

Thank you for making these revisions - I look forward to seeing the published protocol and, in due course, reading the results of the study.
